# Forces, friction and fractionation: Denis Walsh’s *Organisms, agency, and evolution*: 294 pp, Hardcover, ISBN: 1107122104

**DOI:** 10.1007/s10539-017-9585-z

**Published:** 2017-07-27

**Authors:** Andrew Buskell, Adrian Currie

**Affiliations:** University of Cambridge, Cambridge, United Kingdom

**Keywords:** Modern synthesis, Teleology, Metaphysics, Organisms

## Abstract

In Denis Walsh’s *Organisms, Agency, and Evolution*, he argues that new developments in the science of biology motivate a radical change to our metaphysical picture of life: what he calls ‘Situated Darwinism’. The central claim is that we should take the biological world to be at base about organisms, and organisms in a fundamentally teleological sense. We critically examine Walsh’s arguments and suggest further developments.

## Introduction

There is a story—somewhere between myth and history—that philosophers of biology love to tell. It structures undergraduate teaching and organises research agendas. In Denis Walsh’s *Organisms, Agency, and Evolution* (2015), the story serves as a backdrop for a radical reconfiguration of our fundamental metaphysical picture of biology.

The story begins at some point in the early twentieth century, as biologists brought together Darwin’s theory of natural selection with Mendel’s genetic theory of inheritance. This opened the door to a novel mathematical and experimental research agenda in biology: the ‘modern synthesis’. The effectiveness of the synthesis lay in its simplicity, gained by idealising the complexities of biological evolution to isolate a core collection of processes. So, for example, inheritance is to be understood as a process where genes pass on instructions for constructing organisms between generations. Selection then efficiently sorts this information: those instructions which are the most successful in producing viable, fecund organisms are the instructions which multiply.

In this narrative, the main business of synthesis biology centres on explanations for why advantageous traits increase over generational time. Crucially, the synthesis endeavour relied on idealising, abstracting, and black-boxing a number of biological processes. While changes to the germ-line cells matter, changes to the somatic cells don’t. Selection on individuals or genes matters, but selection on populations doesn’t. Natural selection happens when traits are moulded to the environment, not the other way around.

Yet, it is claimed, the beautiful simplicity of the synthesis has come under pressure. When evolution is de-idealised—when those black-boxes are opened—the synthesis vision has faltered. Molecular genetics has not made happy bedfellows with Mendelianism; sequences of DNA have resolutely refused to follow the modern synthesis’s beat (cf. Waters 1994). Development plays a much bigger role picking evolution’s winners and losers than initially supposed. Mutations are more than the roll of a dice; both the source of variation and the variation itself matters. Agent–environment interactions are dynamic and two-directional. The long history of an organism, its phylogenetic placement, and whatever ‘frozen accidents’ have befallen its ancestors, constrain evolutionary fates. Inheritance is no simple gene–gene story: epigenetic, cultural, and ecological factors exert non-trivial influences on inheritance.

Mythic narratives aside, there is an open debate about whether the synthesis is creaking, cracked, or resilient. Despite anti-establishment credentials, phenomena like niche-construction, cultural evolution, evolutionary constraints, and so forth, are often readily incorporated. Indeed, the synthesis happily accommodates new tools, new results, and new phenomena (Wray et al. 2014). The result, we think, is that modern biological theory is unabashedly pluralistic, with an expanding tool-kit, and a sophisticated understanding of the biological world. More to the point, this is the case regardless of whether this expanded tool-kit is part of the same old synthesis, or one that has been revised, radically extended, discarded, or whatever (and, god forbid, regardless of what Darwin actually intended).

Walsh urges us to examine the implications of contemporary biology as a pluralistic, opportunistic enterprise. As he notes, whatever disarray evolutionary theory is in, it is clear that *how* one investigates life has changed. So, setting aside theoretical worries, Walsh focuses on the fundamentals of biology. Given what we know about the machinery of life, what can one say about what *life really is*? That is, given the new riches emerging from empirical work in biology, how should our understanding of the *metaphysics* of biology change?

According to Walsh, such a metaphysics should fundamentally be about *organisms*. Not organisms as one ‘unit’ of selection, or as some replicators’ avatar, but as purposive critters, occupying contexts that they themselves constitute and construct. That is, organisms taken in a fundamentally teleological, agential sense. Walsh’s position—‘Situated Darwinism’—holds that the complexity of empirical work manifest in current biological practice motivates a new metaphysical position, one that puts organisms front-and-centre.

A fair chunk of Walsh’s book (chapters 3 through 6) walks the reader through the synthesis narrative and introduces the empirical challenges sketched above. These developments should be familiar to contemporary philosophers of biology, and so we do not recount them here. However, we do note the fragility that comes with motivating one’s account with historical reflections. Walsh’s views are presented as an alternative to the synthesis myth, and because of this, is hostage to the details of the historical record. Yet whether the synthesis occurred in the manner that Walsh suggests, with the features that he highlights, has been strongly challenged by historians of science (e.g. Milam 2010; Sepkoski 2012).

Nonetheless, we think Walsh’s account of Situated Darwinism is potentially valuable—and can be defended—even if some of the historical details are mistaken. So instead of focusing on these empirical developments or their history, we want to have a crack at explaining the argument of the book, and Walsh’s position, before highlighting some of its gambits. We close by discussing what we think is the main upshot of Walsh’s book, and consider what direction it might take evolutionary theory.

## Situated Darwinism and fractionation

For Walsh, the modern synthesis is shot through with deeply embedded metaphors, analogies, and narratives. These linguistic devices have been sedimented into the bedrock structure of evolutionary theory. Together, they generate a picture of life that Walsh labels *fractionation*. On this picture, living processes—development, inheritance, adaptive change, and the generation of novelty—are conceptually and empirically separated from one another. These processes are in turn identified with proprietary mechanisms. Thus, natural selection is the mechanism of adaptive change; genetic transmission is the mechanism of inheritance.

Fractionation comes in more-or-less concrete forms. Taken instrumentally—as a strategy for grouping together similar entities and processes—twentieth century biology is testament to fractionation’s productivity. But as Walsh cautions, this triumphant history may generate pernicious consequences. When one forgets that fractionation is just an investigative strategy, one is naturally lead to assume that adaptive change *just is* natural selection, the generation of novelty *just is* mutation, and development *just is* the unfolding of the genetic code-script. The pernicious consequences of fractionation come from reading fractionated models into the metaphysics of biology; reifying the convenient and the abstract with the ontological weight of the concrete.

For Walsh, this move makes organisms passive if not downright epiphenomenal. Organisms become (at best) vehicles for genes, battered about by the vagaries of the environment. Situated Darwinism, by contrast, takes organisms as the central business of biology. And organisms are active, intentional. As Walsh writes,

… organisms are not mere objects of evolutionary forces. They are agents of evolutionary change. In pursuing their goals, in negotiating their affordance landscapes, in constructing their conditions of existence, organisms enact evolution (241).

Organisms and environments together generate ‘affordances’: what an organism can do given their form and the environment together. As such, environments and organisms are dual partners—co-constitutors—of the affordances which shape evolution. What does this amount to? Consider moose. Moose range across the arboreal forests of North America and Russia. Their diet largely consists of fresh shoots, forbs, and leaves, which they supplement with sodium-rich aquatic plants. As browsers, their eating strategy is selective: they search for particular foods in their environment, avoiding less desirable fare. Moose are able to be picky in part because of their remarkably sensitive, prehensile lips. These lips aid in distinguishing between fresh shoots and less palatable twigs, in addition to playing a role in bending branches and stripping them of (delicious) leaves.

On a fractionated account, moose have particular phenotypic features—prehensile lips—which benefited their ancestors and led to the flourishing of that trait (more carefully, the germ-line cells that produced that trait in their ancestors) over evolutionary time. Further, the fitness of the prehensile lips was determined by the environment moose were adapting to. Finally, the production of lips was secured by a developmental system that ensured their robust development across generations. Thus, a fractionated understanding of moose lips generates a picture with a clean separation of developmental processes that ensure good moose-lip construction, the germ-line processes of inheritance, and environment–moose interactions which underwrite populations over time.

According to Walsh’s Situated Darwinism, one should emphasize how the lips of the moose, in combination with their forest environment, generates a set of affordances (stripping leaves off branches, for instance), given the goals of the critter in question (minimally, to survive and reproduce). It is these affordances, and the kind of purposive critters that moose are, which both explains moose lips and their interactions with the environment. This teleological explanation might seem topsy-turvy, but Walsh insists the view is a consequence of the emerging truth about the complexity of biology. Let’s examine why.

## Walsh’s argument

Walsh’s Situated Darwinism is—like the synthesis it aims to replace—impressively systematic. Walsh is no mere philosophical tinkerer; he is re-envisioning the theoretical and metaphysical foundations of the discipline. In what follows, we’ll sketch the two main argumentative moves which, we think, underwrites his project. The first concerns the nature of natural selection, the second the locus of biological explanation.

Back in 1984, Elliot Sober famously drew an analogy between physical mechanics and evolutionary theory (Sober 1993/1984). In mechanics, we can understand the movement of objects in terms of their having properties subject to various forces. For example, in curling, two sweepers use brooms to influence the path of the ‘rock’ towards the ‘house’,^1^ their sweeping action reduces friction, which affects the rock’s trajectory. Illegally *burning the rock* involves actual contact between broom and rock. When this happens, the impact of the broom on the rock changes the rock’s trajectory in a mathematically tractable way. Given the rock’s mass, speed and direction, and the broom’s mass, speed, and direction, one can determine how the acceleration and trajectory of the rock will change given the impact. Perhaps natural selection should be understood similarly. Organismic traits in a population—specifically, trait *fitnesses*—are analogous to mass. Natural selection, on this view, is a force which, by acting on fitness, shapes populations. The impact of the broom on the rock influenced the rock’s trajectory; natural selection moves a population towards an adaptive optimum.

For Walsh, this gets things exactly wrong. In the curling case, there is a causal relationship between various forces and the properties of the objects in question. In biology, organisms have various interactions with conspecifics, other critters, and their environments. Animals eat one another, are eaten, mate, and interact with their surrounds in triumphant and tragic ways. They live, die, and reproduce. All of these engagements alter populations—changes which can be *expressed* as being the result of natural selection. Yet, according to Walsh, natural selection is no ‘force’. Instead it is a statistical summary of real causal relations between living, breathing organisms. Unlike a particular event in a curling match, selection is better compared with a statistical summary of Russ Howard’s last few seasons, which picks out general patterns and trajectories over that time. Natural selection is an end-career retrospective, not a particular winning play.

What does this view of natural selection have to do with Situated Darwinism? For Walsh, understanding natural selection as a force leads to a pernicious form of fractionation: the reification of a statistical summary of organismic activity into a concrete, population-level mechanism. Seen in this way, organisms become non-purposive vehicles, tossed upon the sea of the environment.

This is an important critique, one that Walsh has defended across a number of publications (Walsh et al. 2002; Walsh 2007, 2010). But critiquing pernicious fractionation in itself doesn’t motivate Walsh’s Situated Darwinism. We think that weight is carried by what we’ll call the *separability* argument.

In brief, the separability argument blocks the parcelling of life into separate parts, acted upon by distinct forces. One familiar example of such parcelling is a distinction between *internal* and *external* explanation (Godfrey-Smith 1996). *Internal* explanation refers to endogenous properties of the explanandum species, population, or individual. For instance, the kind of organism you are makes a difference to how you will develop, and thus how you might evolve. The vertebrate *bauplan* has been remarkably stable over evolutionary time, and it’s reasonable to think that this has made a difference to what patterns one will see. There are no dragons or other six-limbed vertebrates, because that’s simply not an option available for vertebrates. By comparison, there are multiple ways in which vertebrates might evolve wings, and natural selection has ensured that, under the right conditions, environments conducive to wings have conspired to generate organisms with wings. Here, *external*, environmental influences are central to explanations of life’s forms and trajectories.

Certainly, the internal/external distinction does useful work in explaining the evolvability of organisms (Brown 2014). However, the distinction should not be taken as metaphysical gospel. Indeed, as Walsh underscores, any attempt to draw a distinction between an organism and its traits on the one hand, and the environment on the other, will be made a mockery of by the causal complexity of the biological world.

The important lesson from … studies of ontogenetic development, is that the proper development of organisms involves an enormously complex and widely spread system of causes (157).

For Walsh, because of this complexity, to understand life we need to take on board *everything at once*:

In a nonseparable system, each behaviour is *jointly* caused by the complete suite of its components. We cannot generally attribute specific effects, or specific differences in effect, to specific components of the system. (158)

Why, in order to understand a complex, interrelated system, are we forced into this extreme holism? One can admit the prevalence of feedback, that causes are jointly required to produce effects, and may be highly context-sensitive, but this does not entail that such causes are irrevocably tangled, irreducible tissues. Indeed, such holism jars uncomfortably with Walsh’s own, repeated invocations of modularity (see esp. ch. 7).

Holism is central to Walsh’s push for organism-centred biology. Nonseparability supposedly grounds this holism, and in doing so, places organismic wholes front-and-centre. Yet while the complexity of organisms might, as followers of developmental systems theory (DST) propose, lead one to buy something like causal democracy—that no particular causal component is more important than another (e.g. Oyama et al. 2001)—it may not force one to take organisms as central figures. Indeed, while Walsh argues that organisms are the only things that can “… assimilate, integrate and orchestrate the causal contributions from genes, epigenetic structures, tissues, organs, behaviour and the physical, ecological and cultural setting” (156), in the next section, we’ll question whether this is tenable.

## Scale and teleology

Two distinctive features of Walsh’s position are, on the one hand, *organism*–*centrism*, and on the other, *teleological fundamentality.* In this section, we’ll raise a worry about the latter, and then the former.

Many philosophers and biologists might be suspicious of Walsh’s desire to locate teleology at the centre of biology. Teleological explanations account for events, processes or properties in terms of their purposes. We might say, for instance, that moose have prehensile lips *because* it makes them better browsers. Yet it’s often claimed that irreducible teleological explanations were cast out by Darwin. Nowadays, ‘Darwinian reductionists’ (here meaning reductionists about teleology in particular) would say that moose have prehensile lips because that trait is heritable, and rendered their ancestors more capable of survival and reproduction. Evolution explains the emergence of purpose-likeness from a purely mechanistic basis. Yet, Situated Darwinism resuscitates teleology, positing “… organismic agents as primitive” and thus makes “a commitment to emergence” (217). Teleological purposes are “theoretically indispensable” (218).

We doubt that Darwinian reductionism and Situated Darwinism need be at loggerheads. Evolutionary explanations do not *eliminate* purpose, merely reductively explain them; they allow for purpose in nature, but deny its fundamentality. This seems commensurate with much of what Walsh writes:

Being a goal is not a mysterious intrinsically normative property of a state of affairs. It is a complex relational property, the property of being a state that a goal-directed process tends to attain and maintain (195).

A moose-goal might be to eat only the most succulent leaves, and in virtue of moose environments and their prehensile lips, they have affordances to do so. This state of affairs is maintained because of such robust, ‘goal-directed’ processes. But the reductive evolutionist has machinery to explain why such processes are not ‘mysterious’. That is, they can explain how the processes have come about: they are outcomes of evolutionary processes.

Walsh’s use of teleology does not include an origin story. In fact, it is more-or-less synchronic, and descriptions of how organisms achieve goals is cashed out in cybernetic-like terms. This is in contrast to the Darwinian reductionist and fractionated take on teleology which is interested in such diachronic origin stories. While Walsh’s account focuses on the feedback and regulation conducive to self-regulating organisms, the reductionist is interested in those processes which leads to the emergence of such regulative systems in organisms over generations.

Metaphysically speaking ‘emergence’, ‘primativeness’ and ‘indispensability’ typically imply inexplicability: a primitive posit generates explanatory dividends without itself being explained. But Walsh’s primitivism does not seem to imply such inexplicableness. As such, it remains to be seen whether his appeal to ‘fundamental’ teleology in fact strikes a blow to, or is in tension with, Darwinian reductionism.

Now, to organisms. Walsh rejects fundamentality as residing either at the population-level, with the summaries of natural selection, or at the micro-level, with the idealizations of gene-centrism. Instead, the proper place to look is *in the middle*: where organisms reside. And what counts as an organism is central: they are Walsh’s metaphysical centrepiece, and distinguish him from the less metaphysically inclined DST. Where other philosophers, sensitive to empirical developments in biology have argued for causal democracy against the tyranny of the gene (Oyama et al. 2001), Walsh argues that researchers need a new dictator: the organism.

We worry, however, that Walsh’s organism-centrism hinges on contingent assumptions, particularly concerning the scale at which one works. Indeed, the generality of Walsh’s definition of an organism as a “self-regulating, self-forming system” (157) suggests that organisms can be found at various scales and grades. Though Walsh’s examples are sundry, common-sense critters—cilia, porpoises, and such—his definition admits a far wide set of entities.

Let’s begin at the population-level. Here there are many examples of things that are self-regulating and self-forming. Some of these are subject to debates pertaining to their individual-hood: eusocial insect colonies, slime-moulds, and so forth. Beyond such superorganisms, there are less obvious candidates for Walshian organismality. According to community ecology, for instance, collections of predators and prey maintain population-level properties (stable species-diversity, for instance) in virtue of trophic interactions, diversity, and other features properly attributed to the community (Sterelny 2006). Potentially, Walsh’s category of ‘organisms’ includes not only superorganisms, but communities, ecosystems, and lineages.

In a revealing footnote, Walsh modifies his account of organismality, saying “… an organism is more like a maximal aggregate of such units. Each organism will have among its parts other self-building, self-making, self-repeating systems” (footnote 8, 157). If this is right, then it may be organisms all the way down for Walsh, and possibly, all the way up as well. It’s not obvious to us why the ‘maximal aggregate’ concerning moose is a single moose, as opposed to a population of moose, or a lineage of moose. When one notices the possibility of other ‘maximal aggregates’, Walsh’s apparent focus on common-sense organisms becomes less convincing.

There are several moves open at this point. Walsh might attempt to restrict what it takes to be a ‘maximal aggregate’ such that it picks out the scale of everyday organisms. Alternatively, he might embrace a promiscuous organism-pluralism, where organismality occurs in different amounts at different scales. We worry that this second strategy might be unable to sustain Walsh’s focus on organisms as they are commonly understood. It’s one thing to say that organisms are central to biology, and another to say that ‘organisms’, understood in an idiosyncratic way, are central to biology. Perhaps Walsh’s ‘organism’ talk should be jettisoned altogether in favour of, say, ‘teleological units’. However, this shrinks the distance between Walsh’ Situated Darwinism and metaphysical brands of Darwinian reductionists (who also tend to be relaxed about what constitutes an ‘organism’): what was supposed to be a systematic metaphysical difference starts to look like a difference in emphasis.

## Metaphysics and biology

As we’ve stressed, Walsh’s book is exciting because of its ambitious, unapologetic metaphysical tack. But this is also unusual. This is because philosophers of science tend to be modest in their metaphysical aspirations. While the field is no longer wholly sceptical or hostile towards metaphysics, the typical strategy for making metaphysical claims remains humble. The positive contribution that philosophers tend to make to ontology comes by clarifying the nature and structure of the metaphysical commitments at work in cutting-edge science (Ladyman and Ross 2007). In this way philosophers of biology have helped to make sense of biological individuals (Clarke 2013), group-selection and altruism (Okasha 2006), and homology and analogy (Currie 2013). Though modest, such metaphysical analyses do real work. Philosophers make contributions to science not only by identifying and clarifying metaphysical commitments, but also by making non-trivial contributions to empirical research: they sift complex issues, highlighting unresolved problems and locating areas of productive empirical friction.

Walsh doesn’t adopt this modest strategy. Situated Darwinism is a bold metaphysical hypothesis about the nature of life itself. So, how does Walsh establish his metaphysical claims? One common strategy is to ground such claims via an inference to the best explanation (hereafter; ‘IBE’): the best explanation of the results of scientific work is the truth of some of its claims (Lipton 2004). The nature and validity of the inferences involved in IBEs are contentious (Novick 2016), yet even being bullish about such a strategy, we are sceptical that it can motivate Walsh’s picture. This is because it isn’t obvious on what grounds we might say that Walsh’s explanation is the best. Walsh mentions, but rarely directly engages with, what we might call *sophisticated fractionationism*: an updated and enriched version of the modern synthesis still committed to the idealising assumptions of fractionation. Lacking in such direct engagement, Walsh’s account is thin on reasons for why Situated Darwinism is more virtuous, explanatorily speaking, than sophisticated fractionation.

One way to rationalise the lack of attention to sophisticated fractionation, and thus to IBEs, is to suggest that Walsh’s metaphysical arguments are secured by another strategy, one harkening back to Kant and the German Romantics. Similar to how Kant argued that fundamental ontological structures of subjectivity are required for empirical knowledge, so too might Walsh be arguing that there is an ontological structure of organismality that underwrites development, robustness, and adaptiveness. This fundamental ontological structure is that of an agent pursuing goals by recognising and acting on meaningful affordances. In other words, there are preconditions for the possibility of biological phenomena, and Situated Darwinism provides an account of these preconditions. Walsh might be putting forward a *transcendental* argument for Situated Darwinism.

Suggesting this transcendental interpretation are a number of unusual (at least for philosophy of biology) nods to Merleau-Ponty, Kant, and Haugeland. That Walsh might be adopting such a strategy is also evident in the many places where he moves from empirical considerations to quite beefy metaphysical conclusions. Consider a number of claims we think are representative of such a transcendental strategy:

Proper development depends upon the capacity of organisms to assimilate, integrate, and orchestrate the causal contributions from genes, epigenetic structures, tissues, organs, behaviour and the physical, ecological and cultural setting. (p. 157)

Evolution is adaptive, because organisms are adaptive, goal-directed systems. […] We need to invoke the capacity of organisms to pursue goals in order to explain the origin of adaptive novelties. (p. 203)

Organisms, as reactive, purposive entities condition and regulate both the conditions in which they live and how the conditions impinge on them. The conditions in which form evolves are a joint project of the organism and its setting. (p. 173)

[…] organisms constitute and hold in place the conditions for evolution. (p. 247)

A transcendental reading of Walsh takes him to be arguing that explanations of the development, robustness, and adaptiveness of organisms entails a metaphysical picture of organisms as agents that pursue goals by recognising and acting on meaningful affordances.

It is worth noting that transcendental arguments are hard to motivate: they require showing that empirical facts of the matter *require* there being a particular metaphysical structure. For Kant, the forms of space and time were necessary preconditions for empirical access to the world—for knowledge itself. Yet it is unclear whether Situated Darwinism can appeal to the same strong modal force. Here too, sophisticated fractionation serves as a useful contrast. To the extent that sophisticated fractionation serves as a plausible metaphysical alternative, then it is unclear that one needs Situated Darwinism to make sense of life itself.

Nonetheless, the metaphysical picture produced by Walsh is impressive. And we think his synthetic metaphysical picture is one that many practicing biologists, ecologists, geneticists, and philosophers would agree with. At bottom, organisms do seem to be goal-directed entities that robustly achieve desired ends. Further, aside from being intuitive, the metaphysical picture is congenial and consistent with empirical research. Walsh notes in several places where his metaphysical picture can be idealised in ways to connect it up with fruitful lines of research. Autopoiesis and agency comes up for a brief dialogue here (pp. 194–195), but the main links are to evo-devo (ch. 7) and population genetics (ch. 10). Finally, insofar as the metaphysical picture is intuitive and congenial, it can serve as a platform for conversations between researchers, who can discuss how their empirical approach selectively highlights certain organismic features while bracketing-off others.

A further important benefit of Walsh’s metaphysical stratagem deserves to be mentioned. As we’ve suggested, Situated Darwinism can work as a corrective for those who take fractionation *qua* metaphysical picture too far. Misperceptions, such as the idea that biology is at base *only* about genes, may be pernicious in part because we lack an alternative vocabulary to talk about such phenomena. Situated Darwinism provides just such an alternative vocabulary. In offering a coherent and powerful alternative vision, Situated Darwinism may be opening up some much needed metaphysical breathing room for alternative understandings of life.

Yet there are risks to Walsh’s metaphysical focus. For one, we question the extent to which Walsh’s view generates empirical research. It is one thing to suggest that a metaphysical picture is congenial to and consistent with contemporary scientific work—but generally philosophers of science expect our metaphysics to be geared to knowledge production and justification. Yet as we noted above, little of Walsh’s book is dedicated to showing how Situated Darwinism can aid scientific work (though we suggest how in the next section).

So a pressing worry for Walsh comes down to whether the metaphysics really matter. Assume with Walsh that fractionation is a misleading metaphysical picture. And with Walsh let us agree that fractionation is incredibly successful. If all this can be taken for granted, then the fundamental nature of biology appears to play very little role in the actual science of living nature.

We suspect some of the concerns about the relevance of Walsh’s picture for empirical work can be allayed. However, it requires downplaying the emphasis on metaphysics. Instead of thinking of Situated Darwinism as a metaphysical project, perhaps it should be thought of as a kind of *organizing framework*.

## Extending the view

Situated Darwinism takes risks by making substantive claims about the biological facts of the matter—what evolution *really is*. But one can potentially isolate another, less risky strategy at play in Walsh’s book, one where the metaphysics of biology is less important. Emerging towards the end of the book, Walsh sketches what evolutionary theory would look like when seen through the lens of goal-directed, affordance-utilising critters. So this second, less onerous strategy focuses on the potential of Situated Darwinism as a framework for organising empirical research.

Evaluating organisational frameworks involves considering issues and trade-offs that operate at a very high level of abstraction. For Walsh, two key issues are the extent to which a framework can (a) accommodate the diversity of empirical work being produced by various branches of the biological sciences, and (b) the extent to which it can unify and organise these into an illuminating and empirically fruitful body of theory. Unsurprisingly, he thinks that Situated Darwinism will outperform the Modern Synthesis on these two key desiderata.

This positive alternative takes shape in the final section of the final chapter. There Walsh argues that Situated Darwinism affords an accommodating framework where “any process that contributes to the change (or stasis) in the origin or the frequency of intergenerationally stable forms is potentially an evolutionary process” (p. 241) More than this, Situated Darwinism provides unifying categorisations of such processes *in virtue* of the role they play in helping or hindering organismic goals.

Taking Situated Darwinism as a framework can help to make sense of why it might not have an empirical agenda all its own. It doesn’t highlight new phenomena, or provide new empirical methods. But this does not exhaust the potential benefits of Walsh’s picture: the payoff of Situated Darwinism may lie in its capacity to accommodate and unify multiple traits that operate across multiple spatiotemporal scales. It does so by highlighting gross functional similarities among such traits—functional similarities that only become visible when seen through the lens of goal-directed agents.

Take the category Walsh calls ‘buffering’. This lumps together the activity of immune systems, behavioural strategies for thermoregulation (such as migration), counteractive niche construction (Odling-Smee et al. 2003), developmental robustness, and phenotypic accommodation. A motley crew to be sure. Yet Walsh argues they can all be unified in virtue of being “various activities of organisms [that] can buffer the effects of mutations and environment on form.” (p. 241). That is, all of these different character traits provide some way of keeping organisms within a viable range of conditions. Similar high-level functional roles underpin Walsh’s other categories: stabilising, innovating, facilitating, and co-opting (pp. 242–246).

As Walsh admits, “Situated Darwinism is nascent, inchoate, struggling for a definitive articulation. No doubt it will take a considerable amount of time to grow, and it will change along the way, if it survives its infancy at all.” (p. 231) The positive story we have suggested—where Situated Darwinism serves as a framework for unifying and organising biological work—provides good reasons to foster this growth, and to make sure it survives infancy.

## Footnotes

1 The ‘rock’ being the puck-like ‘ball’, the ‘house’ being the scoring zone.
